# Opportunities and Challenges Surrounding the Use of Wearable Sensor Bracelets for Infectious Disease Detection During Hajj: Qualitative Interview Study

**DOI:** 10.2196/60484

**Published:** 2025-04-08

**Authors:** Noha Maddah, Arpana Verma, John Ainsworth

**Affiliations:** 1 Division of Informatics Imaging and Data Sciences, School of Health Sciences, Faculty of Biology, Medicine and Health Manchester Academic Health Science Centre, Centre for Health Informatics The University of Manchester Manchester United Kingdom; 2 Department of Health Services and Hospitals Administration Faculty of Economics and Administration King Abdulaziz University Jeddah Saudi Arabia; 3 Division of Population Health, Health Services Research & Primary Care, Faculty of Biology, Medicine and Health Manchester Academic Health Sciences Centre The University of Manchester Manchester United Kingdom

**Keywords:** wearable sensor, unified theory of acceptance and use of technology, task-technology fit, hajj, presymptomatic detection, infectious diseases, artificial intelligence

## Abstract

**Background:**

Wearable sensor bracelets have gained interest for their ability to detect symptomatic and presymptomatic infections through alterations in physiological indicators. Nevertheless, the use of these devices for public health surveillance among attendees of large-scale events such as hajj, the Islamic religious mass gathering held in Saudi Arabia, is currently in a nascent phase.

**Objective:**

This study aimed to explore hajj stakeholders’ perspectives on the use of wearable sensor bracelets for disease detection.

**Methods:**

We conducted a qualitative, theoretically informed, interview-based study from March 2022 to October 2023 involving a diverse sample of hajj stakeholders, including technology experts, health care providers, and hajj service providers. The study was guided by the task-technology fit model and the unified theory of acceptance and use of technology to provide a comprehensive understanding of the factors influencing the acceptance and use of the technology. Semistructured in-depth interviews were used to capture perspectives on using wearable sensor bracelets for infectious disease detection during hajj. Thematic analysis of interview transcripts was conducted.

**Results:**

A total of 14 individuals were interviewed. In total, 4 main themes and 13 subthemes emerged from the study, highlighting crucial challenges, considerations, recommendations, and opportunities in the use of wearable sensor bracelets for the presymptomatic detection of infectious diseases during hajj. Implementing wearable sensor bracelets for disease detection during hajj faces obstacles from multiple perspectives, encompassing users, implementing stakeholders, and technological factors. Hajj stakeholders were concerned about the substantial financial and operational barriers. The motivation of implementing stakeholders and users is essential for the acceptance and uptake of devices during hajj. Successful integration of wearables into the hajj surveillance system depends on several factors, including infrastructure, device features, suitable use cases, training, and a smooth organizational integration process.

**Conclusions:**

This study provides valuable insights into the potential opportunities and challenges of adopting wearable sensor bracelets for disease detection during hajj. It offers essential factors to consider and important suggestions to enhance comprehension and ensure the effective implementation of this technology.

## Introduction

### New Generation of Digital Tools for Public Health Surveillance

Understanding and reacting to health trends affecting communities is a core function of public health systems [1]. Public health surveillance is the tool used to respond to these health trends through “the ongoing, systematic collection, analysis, and interpretation of health-related data essential to planning, implementation, and evaluation of public health practice” [1]. The COVID-19 pandemic has brought to light the urgent need for real-time public health surveillance to enhance the use of evidence in decision-making [1]. The 2020 Riyadh Declaration was formulated as a response to the global pandemic, aiming to propose a series of suggestions that would effectively tackle the shortcomings observed in global public health response systems. The Riyadh Declaration emphasized the significance of implementing digital health solutions that are both scalable and sustainable, as well as the adoption of health intelligence [1].

Traditional public health initiatives targeted at preventing the spread of infectious diseases such as the recent COVID-19 have limitations that digital health technologies could address [2]. The World Health Organization has defined digital health technologies as “the field of knowledge and practice associated with the development and use of digital technologies to improve health” [3]. The use of digital health tools during the global pandemic has exhibited their efficacy in fighting, mitigating, and controlling the transmission of emerging infectious diseases [2]. There is a significant potential for adopting digital surveillance as a valuable tool in monitoring and controlling infectious diseases [4]. Internet of Things (IoT) technological advancement has proven its ability to augment and optimize conventional preventative public health strategies and methodologies [2]. These internet-based technologies possess certain characteristics that enable the expedited detection of infectious diseases [4]. The term IoT pertains to a network of interconnected objects, devices, and systems that engage in electronic communication and receive, process, and send digital data with minimal human involvement [5]. IoT is expected to reach its “plateau of productivity” during the next 5 to 10 years [5].

Detection is the initial and important aspect in preventing infectious diseases [5]. In the context of these diseases, the computation speed plays a crucial role in reducing the time required for their diagnosis, facilitating prompt implementation of preventive measures. The performance of system automation has been found to enhance surveillance’s efficacy in widespread disease transmission. In addition, it has proven to be an effective means of reducing the workload of medical workers during pandemics [4].

### Wearable Technologies and Body Sensors

Wearable technologies are integral to widespread IoT technical advancements [6]. Wearable technologies are intelligent electronic devices worn on the body to measure, analyze, and transfer information. The data may encompass many bodily indicators associated with essential physiological parameters and levels of physical exertion. These wearables have the capability to provide information to the user by means of display technology or vibrotactile feedback [7].

Wearable sensors have garnered considerable attention in the past 10 years, with a particular focus on their application within the health care sector [8,9]. Their ability to establish connections with other devices and the IoT, facilitating data exchange, has led to their designation as “smart” devices, setting them apart from traditional, nonconnected step counters, wristwatches, or clothing [10]. Wearable sensors have the capability to monitor, collect, and send data [6]. They offer precise, credible, and real-time data regarding an individual’s behaviors and activities, hence aiding users in identifying potential issues. Wearables collect raw data from measurements using sensors to store them and use them for things such as performance evaluation and ongoing health monitoring [11]. These sensors can be applied in several areas, such as illness monitoring, diagnosis and treatment, and health management [12].

Various terms are used to refer to wearable sensor devices that capture and analyze real-time physical data in a nonintrusive manner. These terms encompass *smartwatches* [9,13], *smart sensors* [14], *wearable activity trackers* [15], *wearable sensors* [16], *wearable physiological sensors* [17], *integrated biosensors* [18], and *sensor bracelets* [19]. In the context of this research, the phrase “sensor bracelet” will be used.

Extensive research has been conducted on wearables to investigate their potential in health monitoring. However, there is a noticeable trend in current research toward applying wearables for the presymptomatic detection of infectious diseases [9,19]. This transition signifies the growing importance of early detection of diseases, especially within the context of public health and large-scale occurrences. The use of wearable technology has emerged as a significant factor in this pursuit, presenting the possibility of identifying infections before the manifestation of symptoms. This capability facilitates prompt interventions and preventive actions. The increasing emphasis on this matter highlights the significant contribution that wearables can make in augmenting health care and methods for disease control.

Wearable devices (sensor bracelets) have gained attention for their ability to anticipate both symptomatic and presymptomatic identification of viral respiratory infections by sensing changes in physiological markers, as highlighted in the recent literature [19,20]. Mitratza et al [16] conducted a systematic review to assess the value of a range of wearable sensor devices in detecting COVID-19. In their review, they noted that, while wearable devices show promising potential for detecting COVID-19, the findings are still in the early stages. They emphasized the need to consider how differences in sensor methods, data processing, and algorithms affect the detection of infection-related changes in physiological measurements, as well as the importance of addressing sources of bias. Building on this, Risch et al [19] assessed the use of a smart bracelet in the early detection of changes in physiological parameters related to COVID-19. With a low false-positive rate, their wearable sensor bracelet algorithm was able to identify 71% of COVID-19 cases up to 2 days before symptoms manifested. This demonstrates the efficacy of using wearable technologies for the early detection of diseases. Mishra et al [9] conducted a study whereby they demonstrated the use of data from smartwatches to detect respiratory infections such as COVID-19 in real time within a large-scale scenario. The study identified 63% of COVID-19 infections with a low false-positive rate. Furthermore, Hirten et al [2] evaluated the longitudinal collection of heart rate variability (HRV) metrics from a wearable device for the prediction of COVID-19. Their study indicated that HRV data from the Apple Watch can predict COVID-19 and identify symptoms. In addition, alterations in HRV were observed before a positive polymerase chain reaction test, indicating its potential for early infection detection.

### Hajj, the Largest Annual Religious Mass Gathering, and Disease Transmission

Safety and security have consistently been of utmost importance within the framework of hajj, a significant religious event that attracts participants worldwide. This annual massive religious gathering in Makkah, Saudi Arabia, attracts almost 3 million pilgrims from 180 countries. These pilgrims spend several weeks in the cities of Makkah and Madinah. The problem of overcrowding and the lack of compliance with government-mandated preventative measures for diseases have been highlighted in several previous studies [17,21-23]. This lack of compliance contributed to a long-standing high occurrence of respiratory infections before the COVID-19 outbreak. Low adherence among pilgrims has been attributed to a number of factors, including insufficient coverage and limited access to information [24].

Using intelligent and comprehensive monitoring tools for participants in this mass event is crucial to reducing the risk of future tragedies. Saudi Arabia uses several technical methods such as spatial computing, crowd simulation, mobile apps, and big data analytics for hajj activities [25].

In recent times, there has been an increasing focus on incorporating state-of-the-art technologies to tackle the distinctive difficulties linked to hajj. The hajj pilgrimage, characterized by its large-scale congregation of millions of pilgrims, poses significant logistical, health, and safety complexities. Disease surveillance has emerged as a crucial component of hajj management, progressively evolving over time by assimilating insights gained from previous operations.

The Kingdom of Saudi Arabia has been at the forefront of implementing digital solutions to improve the pilgrimage experience as part of the national strategy and the 2030 vision. One of the notable technological developments in this context involves the application of the smart bracelet initiative, which involved the collaboration of multiple governmental authorities. In 2021, the Pilgrims’ Smart Bracelet initiative was introduced, offering 5000 pilgrims bracelets equipped with data storage and transmission capabilities, thus contributing to the IoT. The main purpose of the bracelet was to provide extensive information on the pilgrim, including their health condition. The device enabled the tracking of important health indicators such as blood oxygen saturation and pulse rate while also offering functionalities for requesting immediate medical or security support, guaranteeing swift reaction and help. In addition, pilgrims were given awareness messages via the bracelet they wore. The initiative’s implementation entailed incorporating and enhancing business models and systems, spanning both operational and technological facets of the project [26].

The practice of postmortem detection for infectious diseases underscores the criticality of real-time data and analytics to avert their transmission, which has severe social and economic repercussions for citizens worldwide [5]. Within the framework of hajj, it is imperative to not only identify diseases before symptoms appear but also find and follow the individuals who have been in contact with infected individuals. This requires incorporating tracking elements into the device. The current state of affairs presents an opportune environment for scientific inquiry specifically aimed at recognizing and addressing nascent hazards and difficulties to predict and alleviate them. Hence, this study endeavored to investigate the opportunities and challenges surrounding using wearable sensor bracelets for presymptomatic detection of infectious diseases during hajj.

## Methods

### Theoretical Framework

Technology acceptance models have been criticized for their limited ability to evaluate the extent to which wearable technology offers the requisite functionalities to attain desired outcomes. This concern can be effectively resolved by integrating the task-technology fit (TTF) and technology acceptance models [27].

Studies have shown that integrating the TTF model and the unified theory of acceptance and use of technology (UTAUT) enhances the predictive strength for technology adoption. The TTF-UTAUT model integrates 2 key frameworks to predict technology adoption [28]. The TTF model is defined as the interdependence between individual (a technology user), technology (data, hardware, software tools, and the services they provide), and task (an activity carried out by individuals to produce the required output) characteristics [26]. The UTAUT suggests that the actual use of technology is determined by behavioral intention. The perceived likelihood of adopting the technology depends on the direct effect of 4 key constructs: performance expectancy, effort expectancy, social influence, and facilitating conditions [29].

The UTAUT is considered beneficial for the complex nature of this research, which involves evaluating several interrelated factors in disciplines such as health informatics and public health during large-scale events. The UTAUT can provide insights into the determinants that affect the adoption and use of wearable sensor bracelets among various stakeholders participating in the hajj pilgrimage. To provide a more thorough explanation, the TTF model was integrated with the UTAUT. The TTF model can be used to evaluate the degree to which the features of the technology align with the requirements and demands of its users, including both pilgrims and authorities. This integration improves understanding and focuses on particular elements inside the UTAUT.

**Figure 1:**
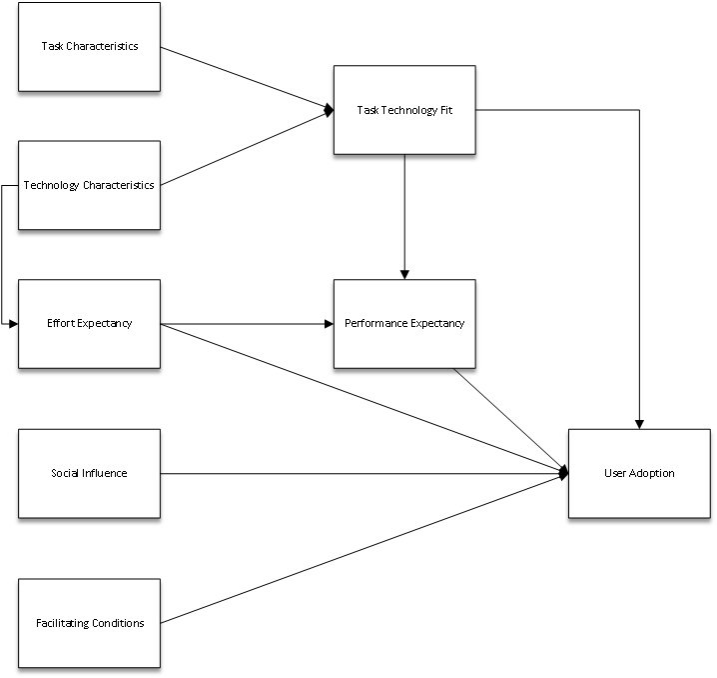
Task-technology fit and unified theory of acceptance and use of technology.

To aid in effectively applying wearable sensor bracelets during the hajj pilgrimage, this integrated approach can offer a comprehensive understanding of stakeholders’ viewpoints. Figure 1 [28] presents the model used.

### Study Design

We conducted in-depth, semistructured, one-to-one qualitative interviews from March 2022 to October 2023 with 14 selected hajj stakeholders, organizers, and professionals working in the field to capture their perspectives on using wearable sensor bracelets for infectious disease detection during hajj.

### Data Collection and Sampling Strategy

#### Overview

A nested cohort approach was used to explore stakeholders’ perspectives [30]. Participants were identified through purposive sampling and snowball sampling techniques. Purposive sampling is a method used to gather information and gain a comprehensive understanding of a certain topic of interest. This methodology entails the selection of data cases based on their capacity to yield data that are rich in information for comprehensive analysis [31].

A purposive sampling strategy was used to identify participants who were directly involved in the smart bracelet initiative. This resulted in a core group of 4 participants who held senior positions within the organizational hierarchy. The key stakeholders were identified through the organizational website and were reached purposefully via email. These individuals offered comprehensive perspectives on the initiative as a result of their direct expertise and authoritative decision-making positions. Concurrently, a more extensive cohort of 10 participants selected using snowball sampling was incorporated to gather insights into the general use of technology during hajj, specifically from individuals who directly interacted with pilgrims. Although these individuals were not directly involved in the smart bracelet initiative, their contact with pilgrims offered vital contextual information. We reached out to these participants directly via their contact numbers.

The inclusion and exclusion criteria for participants are detailed in Textbox 1. This study adhered to the guidelines outlined in the COREQ (Consolidated Criteria for Reporting Qualitative Research) checklist [32].

**Inclusion and exclusion criteria for participants in this study.**

**Inclusion criteria**

- Individuals in senior positions within the organizational hierarchy who were directly involved in the smart bracelet initiative, including those with technological, medical, organizational, or logistical expertise
- Individuals who have experience and are present in service organizations during hajj (eg, *tawafa* organizations) and can offer their perspective on the technologies used and how pilgrims interact with or accept them

**Exclusion criteria**

- Individuals who are not directly involved in the smart bracelet initiative or do not occupy senior positions
- Individuals who lack pertinent experience or insight into the adoption and use of technology by pilgrims during hajj.

#### Interviews

We used a systematic approach to engage with our potential participants before the interviews started. The interview questions were then given to participants who replied favorably and indicated their desire to take part. This action guaranteed that participants had enough time to prepare for the subsequent interview, promoting a deeper and more thoughtful discussion. We then scheduled the interviews after allowing participants to receive the interview questions and prepare.

#### Interview Topic Guide

The interviews were guided by a semistructured topic guide exploring professional background, experience, and involvement with the 2021 Pilgrims’ Smart Bracelet initiative. Questions were based on existing literature and organizations’ online information to understand the current capabilities and existing tools used during hajj in addition to opportunities, gaps, and challenges associated with implementing wearable sensor bracelets for infectious disease detection during hajj. Questions were tailored to the specific characteristics of participants and evolved in light of emerging findings. We explored the advancements of existing tools and technologies used during hajj for public health surveillance, their limitations, and stakeholders’ vision and perceptions regarding using wearable sensor bracelets for disease detection during hajj.

The interview questions for key stakeholders who were directly involved in the smart bracelet initiative primarily addressed the capabilities of the surveillance system, the technologies and tools used during hajj to support the event, their opinions on wearables for disease detection, and their insights into the smart bracelet initiative. In contrast, interviews conducted with participants who had minimal involvement in the smart bracelet initiative but were highly involved in assisting pilgrims focused on their experiences and viewpoints regarding the acceptability of the latest technology deployed during hajj by the pilgrims. A copy of the topic guide can be found in Multimedia Appendix 1.

Digital audio recorders with encryption were used to record the interviews. Interviews were transcribed verbatim, read numerous times, and annotated to fully immerse us in the facts.

### Data Analysis

Thematic analysis of interview transcripts was conducted [31]. This study used an inductive thematic analysis to investigate participants’ experiences and perceptions regarding the use of wearable sensor bracelets for disease detection during hajj. The UTAUT and TTF framework variables served as a guide for the interview questions, but themes were allowed to naturally arise from the data. In the subsequent analysis, a deductive approach was applied to identify relationships between the emergent themes and the theoretical constructs of the UTAUT and TTF model. This hybrid approach allowed for both the discovery of novel insights and the integration of findings with established theoretical frameworks.

The primary author, NM, led the data analysis by integrating the UTAUT [33] and TTF models [34] to create a comprehensive framework. This framework posits that the user’s intention to use technology and their acceptance of it are influenced by many criteria derived from the UTAUT as well as the degree of compatibility between the task at hand and the technology being used [28].

In this analysis, we explored the use of wearable sensor bracelets for disease detection during hajj. We thoroughly evaluated several aspects such as the integration of technology, development of systems, augmentation of infrastructure, and acceptance among users and suppliers. This comprehensive exploration allowed us to understand the intricate interplay of factors influencing the successful deployment of wearable sensor technology within the unique context of hajj.

Upon completing data collection in our study, we promptly commenced the analysis process to identify any significant findings.

We eventually arrived at the realization that we had obtained enough data to attain data saturation when we noticed that the same subjects kept cropping up during our observations of the data-gathering process and no new ones were introduced. After completing the data collection and analysis, it became evident that no new themes were developing. This confirmed that we had achieved a state of saturation in our findings.

To improve the dependability and verification, synopses of discoveries were regularly communicated to other researchers engaged in the study subsequent to each interview with a participant. The completed transcripts and field notes were systematically arranged and categorized in NVivo (version 12; QSR International) using researcher-derived codes (latent codes) obtained from the interview guide. We followed the characteristics of an inductive coding strategy to guarantee thoroughness and complexity in our investigation [31].

### Ethical Considerations

According to the ethical guidelines set forth by the University of Manchester, this research study did not require independent ethical review and was categorized as low-risk research (“Work with professionals; including interviews, focus groups, questionnaires, etc” [35]). All study data were fully anonymized, with no personal identifiers collected, and were securely stored to ensure privacy and confidentiality in accordance with the University of Manchester data protection guidelines. We initially sent a participant information sheet to each potential participant via digital communication tools informing them of the study’s purpose and why they had been selected. Participation was voluntary, and no compensation was offered or required.

## Results

### Participant Characteristics

A total of 14 semistructured, in-depth interviews were carried out, with sessions having a duration ranging from 20 to 80 minutes. The mean duration of the interviews was 32.0 (SD 20.8) minutes. The variation in interview durations can be ascribed to the participants’ varying levels of expertise and knowledge and the degree of their engagement with the smart bracelet initiative. Individuals that had greater direct engagement with the initiative generally offered more comprehensive perspectives, leading to lengthier interviews. In contrast, individuals with limited direct experience or engagement generally had shorter interviews.

The interview participants in our study encompassed a wide array of backgrounds, which included individuals involved in hajj, specialists in public health, and professionals from several disciplines (Table 1). A subset of the individuals involved in the study held prominent positions within governmental entities, whereas others held positions with varied levels of decision-making power.

**Table 1:** Study participants’ expertise and sectors.

Participant number	Expertise	Sector
1	Systems engineering, business, and project management	Hajj and *umrah* services
2	Health care provider and public health specialist	Health care and public health
3	Health care provider and public health specialist	Health care and public health
4	Communication engineering and artificial intelligence	Technology or consulting (business)
5	Service provider	Accommodation
6	Service provider	Accommodation
7	Service provider	Accommodation
8	Service provider	Accommodation
9	Service provider	Guidance to holy sites
10	Service provider	Enrichment (educational and awareness)
11	Service provider	Arrivals and departures
12	Service provider	Arrivals and departures
13	Service provider	Transportation
14	Service provider	Transportation

In total, 2 distinct groups were formed based on the results of the conducted interviews. The initial cohort consisted of 4 participants who were directly involved in the 2021 initiative and made significant contributions to this study. The second cohort consisted of 10 persons who were not directly involved in the 2021 initiative and had no additional insights to contribute regarding the use of wearable devices during hajj. Nevertheless, these individuals were involved in the provision and coordination of hajj services as well as direct engagement with pilgrims.

Each participant had a minimum of 10 years of professional experience in their respective fields. The individuals who made these contributions were located in Saudi Arabia. A total of 71% (10/14) of the interviews were conducted via telephone, and the remaining 29% (4/14) were conducted using the Zoom platform (Zoom Video Communications). Audio recordings were gathered for all interviews.

### Generated Themes: An Overview

**Figure 2:**
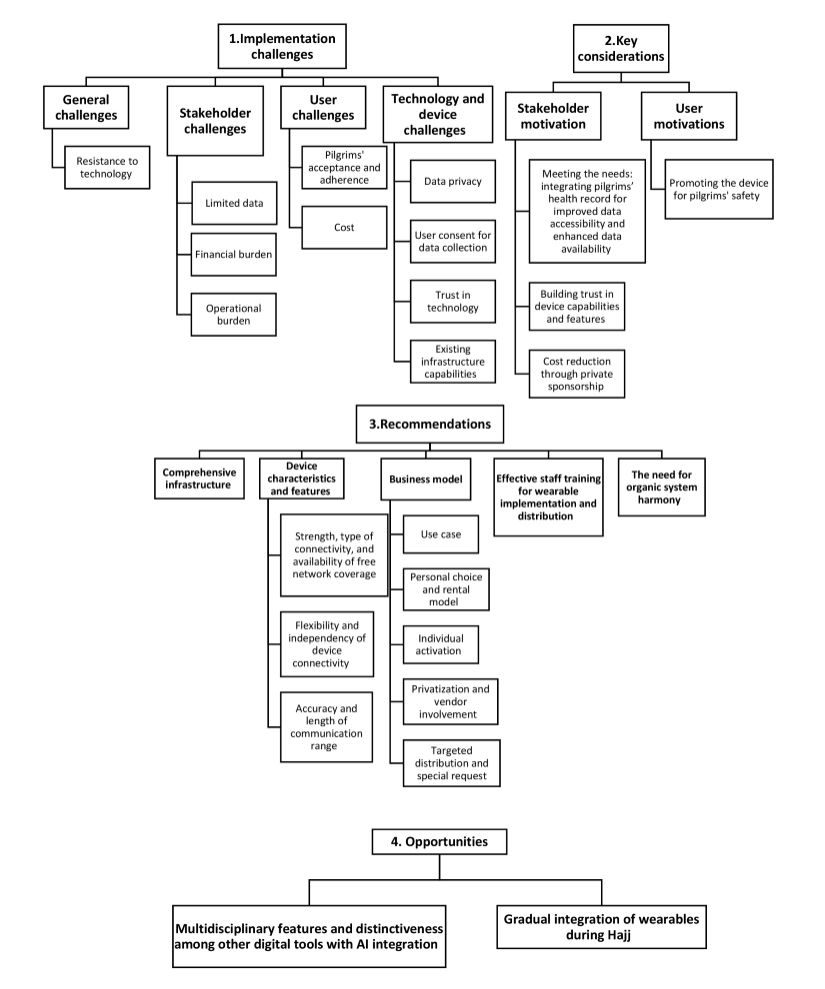
Thematic analysis of the conceptual theme framework of the interviews. AI: artificial intelligence.

The analysis generated insights on 4 main themes and 13 subthemes (Figure 2), highlighting crucial challenges, considerations, recommendations, and opportunities in the use of wearable sensor bracelets for the presymptomatic detection of infectious diseases during hajj. A narrative synthesis was used to report the findings obtained from data collected from the first group of 29% (4/14) of the participants who played a role in the 2021 smart bracelet initiative. Table 2 highlights inputs from each of the 4 participants related to the identified themes.

For the second group, a brief summary of the findings is presented highlighting important observations without a detailed analysis.

**Table 2:** Themes generated during data collection.

Theme and subthemes			Participant			
			1	2	3	4

**Implementation challenges**						
	**General challenges**					
		Resistance to technology		✓		
	**Stakeholder challenges**					
		Limited data		✓		
		Financial burden			✓	✓
		Operational burden	✓			
	**User challenges**					
		Pilgrims’ acceptance and adherence		✓		
		Cost				✓
	**Technology and device challenges**					
		Data privacy	✓	✓		✓
		User consent for data collection	✓			✓
		Trust in technology			✓	
		Existing infrastructure capabilities			✓	
**Key considerations**						
	**Stakeholder motivations**					
		Meeting needs		✓		✓
		Building trust in device features and capabilities	✓	✓		
		Cost reduction through private sponsorship	✓	✓	✓	
		Interest of entities involved	✓			✓
	**User motivations**					
		Promoting the device for pilgrims’ safety	✓	✓		
**Recommendations**						
	Comprehensive infrastructure				✓	✓
	Device characteristics and features					✓
	Business model		✓	✓		✓
	Effective staff training for wearable implementation and distribution				✓	
	The need for organic system harmony				✓	
**Opportunities**						
	Multidisciplinary features and distinctiveness among other digital tools with AI^a^ integration			✓		✓
	Gradual integration of wearables during hajj			✓		

^a^AI: artificial intelligence.

### The First Group’s Perceptions of Using Wearable Sensor Bracelets

#### Overview

The idea of “smart hajj” was a recurring topic in the interviews as respondents emphasized their goals for the future and their current capabilities and constraints. The concept of smart hajj signifies the continuous endeavors of the hajj organization to adapt to technological advancements and improve the overall pilgrimage journey. The concept comprises various technical solutions designed to tackle difficulties and enhance the welfare and security of pilgrims.

However, the deployment of smart hajj and incorporating wearable devices into the technology used by hajj authorities present challenges and potential. Several important points were raised, and suggestions were made by the participants.

#### Implementation Challenges of Wearable Sensor Bracelets for Infectious Disease Detection During Hajj

In the context of using digital health technologies for public health surveillance, participants in our study highlighted a range of challenges in deploying wearable sensor bracelets for disease detection during hajj. The issues encompassed a range of areas, including general challenges, challenges connected to implementing stakeholders, challenges linked to users, and challenges related to devices and technology.

##### General Challenges: Resistance to Technology

Participants voiced apprehensions over the wider difficulties linked to integrating technology such as wearables within the public health domain. They emphasized a vital concern related to resistance from both users and implementing stakeholders due to factors such as data privacy, information security, and human rights:

We will face a challenge in using technology for public health due to data, information, and human rights. This challenge is present, and we will face resistance.

Participant 2; health care provider and public health specialist

##### Stakeholder Challenges: Limited Data, Financial Burden, and Operational Burden

Participants cited concerns regarding various factors from the hajj implementing stakeholders’ perspective, including limited data availability, resistance to adoption, and the operational and financial burdens associated with wearable implementation. In addition, the conversation explored several aspects pertaining to the previous smart bracelet initiative, elucidating significant barriers that hindered its advancement.

The limited availability of health data can be attributed to the apprehensions of pilgrims regarding disclosing their medical records to hajj authorities. There was a concern among individuals that the act of revealing specific information pertaining to their health status and underlying medical disorders would potentially impede their ability to be granted permission to partake in the hajj pilgrimage or, alternatively, affect their eligibility to obtain hajj visas:

Pilgrims worry that if they mention any medical condition, that the Ministry of Hajj and Umrah will not issue them the visa. For example, they do not mention that they have Cancer fearing that they will not get issued the visa. I do not mind if the pilgrim has Cancer, I will treat you, but I need to know in advance to prepare and provide you with the service.

Participant 2; health care provider and public health specialist

One major issue brought up by participants, especially the hajj organizers, was the expense of integrating wearables throughout the pilgrimage. Considering the wide range of services they already offer, they voiced concerns regarding the cost load related to the adoption of wearables. Participants attributed the failure and incomplete implementation of the previous smart bracelet initiative (NUSUK) to the high cost of wearables and the significant budget needed for their deployment. Hajj organizers questioned whether they could cover the expense of integrating wearables for all pilgrims:

Let’s return to the smart bracelet project and why it was not completed. Finance was an issue at that stage, and the projects couldn’t proceed. The cost of these devices is considered high.

Participant 4; communication engineering and artificial intelligence (AI)

Can we afford to provide the wearable to all pilgrims?

Participant 3; health care provider and public health specialist

Participants highlighted the operational challenges associated with implementing ubiquitous sensor bracelets, including the need for staff training and the availability of expert teams for data analysis and decision-making. Concerns were also expressed over the substantial data flow generated by wearables. Despite the complete readiness of the expert teams, there were concerns that these efforts might not produce valuable outcomes owing to a lack of user engagement:

It will be a huge operational load.It needs a specialised team that deals with the data collected by the watch and decides whether the person needs a team to be dispatched. So, there was a big operational load with a minimal value, especially during Hajj.

Participant 1; systems engineering, business, and project management

##### User Challenges

###### Pilgrims’ Acceptance and Adherence

Participants underlined the difficulty of obtaining user acceptance, particularly in light of the wide range of backgrounds, beliefs, and degrees of faith in technology exhibited by pilgrims hailing from >180 nations worldwide. Stakeholders observed that the implementation of the tracking and tracing feature may potentially discourage certain pilgrims from adopting or adhering to the technology:

Once you mention to the user that they can’t take the bracelet off their wrist, or you can’t give it to someone else, cut it, or I will notice what happens to the bracelet once you have done any of these. It will be like you are tracking me as a prisoner, and there will be high resistance.

Participant 2; health care provider and public health specialist

###### Cost

In addition to being a financial burden for the providers, wearable device costs may be seen as an obstacle by users if they are part of their hajj package. Participants voiced apprehension regarding the potential financial burden of including the cost of the bracelet in the pilgrims’ bundles, deeming it to be costly:

If we estimate that the cost of the bracelet will be 100 Saudi Riyal, it will be considered expensive for pilgrims to add that amount to their package.

Participant 4; communication engineering and AI

###### Technology and Device Challenges: Data Privacy and Users’ Consent, Trust in Technology, and Existing Infrastructure Capabilities

The issue of data privacy is intricate and delicate, particularly in relation to the acquisition of user data through diverse technological means. Privacy considerations are important in using wearable sensor bracelets for public health surveillance during events such as hajj. This is primarily due to the acquired data’s sensitive nature and the wide range of users participating in these activities. When individuals’ data pertaining to their health and movements are collected, it is natural for them to express concerns regarding the entities that possess access to these data and the potential applications they may be subjected to:

Well, the issue of wearables usually and all digital solutions are human rights.

Participant 2; health care provider and public health specialist

The interviewees emphasized the difficulties faced while implementing the hajj smart bracelet initiative (NUSUK), specifically focusing on data protection concerns. They underscored the significance of acquiring informed consent for data collection by technological means, such as health data and location information:

Regarding data privacy, no data can be collected without an individual consent. Tracking location and gathering medical or health data as you know are considered private data which fall under the General Data Protection Regulation (GDPR).

Participant 4; communication engineering and AI

Participants expressed certain reservations about the usefulness of wearable technology. One of the difficulties that participants expressed concerns about was the efficacy of wearable devices. This substantially enhances individuals’ confidence in the deployment of wearable sensor bracelets for disease detection during the hajj pilgrimage. In alternative terms, participants emphasized the necessity for these technologies to possess a high level of efficacy to foster a sense of assurance in their use:

There are lots of challenges. First, the technology part. How effective is it, how does it capture data, and is it durable? Is it accurate? I think we still can’t say that the wearables are ready from the technological side.

Participant 3; health care provider and public health specialist

Despite the notable advancements in the network infrastructure built in the Kingdom of Saudi Arabia, participants expressed concerns regarding the adequacy of connectivity strength necessary to effectively manage the vast number of devices used during hajj and the amount of data transmitted:

What if we have 2 million devices in an area with weak connections? Do you think we can manage it? Still early.

Participant 3; health care provider and public health specialist

#### Key Considerations in the Adoption of Wearable Sensor Bracelets for Disease Detection During Hajj

##### Overview

The motives of both consumers and providers significantly impact the successful integration of technology in any domain. In implementing wearable sensor bracelets during the hajj pilgrimage, it is imperative to thoroughly examine and comprehend the motives of all stakeholders and users concerning their acceptance and adoption of this technology. Participants identified several factors that should be considered to encourage stakeholders.

##### Stakeholder Motivation

###### Meeting Needs: Integrating Pilgrims’ Health Records for Improved Data Accessibility and Enhanced Data Availability 

Stakeholders are primarily driven to integrate technology into their strategic plans when it caters to their distinct requirements and eliminates the obstacles they have experienced. Furthermore, stakeholders tend to adopt a technology when it effectively mitigates current challenges and when the characteristics and capabilities of the device correspond to their specific needs. The motivation behind this behavior arises from a comprehensive understanding of how the technology may optimize their activities and contribute to their overarching goals.

The availability of health data, especially with the heterogeneity of languages of pilgrims attending hajj, is of tremendous value to health care professionals as it helps them determine the individual requirements of participants (hajj attendees) and make required preparations. Implementing a consolidated health data system would significantly streamline the identification and understanding of the medical background and requirements of individuals regardless of the health care facilities they visit within the holy sites and Makkah hospitals. Integrating systems would facilitate uninterrupted access to participants’ records and optimize the delivery of critical health care services:

Ministry of Health needs to have a complete EHR for the pilgrim, they would prefer to have reliable and accurate health data, so data availability.

Participant 4; communication engineering and AI

Today, I need basic information. Wearables for public health are different from normal bracelets. For the normal bracelet, I need major clinical data. For example, I need to know in advance the conditions that the patient (pilgrim) has, such as if he has kidney failure, if she is pregnant and so on, so I get ready to serve them. I do not want to be surprised; I want to be ready.

Participant 2; health care provider and public health specialist

###### Building Trust in Device Capabilities and Features

From the perspective of health care service providers, participants emphasized the key features that must be incorporated into wearable sensor bracelets to instill faith in and reliance on these devices, hence minimizing the necessity for additional evaluation. The accuracy of the data collected by these devices is a crucial factor. Although health care providers may choose to perform supplementary evaluations such as laboratory tests, it is imperative that the wearable device delivers data of utmost precision. The significance of accuracy cannot be overstated as it guarantees the reliability of the obtained information and establishes a robust basis for further evaluations:

If you look at Apple Fit watch or any other Fitbit watch, the heart rate increases if a person makes any physical effort. Sometimes even if you are sitting, your heart rate could be irregular.

Participant 1; systems engineering, business, and project management

In addition to recognizing the significance of trust and the attributes of the wearable sensor bracelet, participants underscored their preference for specific aspects of wearable technologies. A further motive emphasized by the study participants pertained to a particular attribute of the device, namely, its capacity to both transmit and receive data. Participants emphasized the significance of bidirectional data connection as it enables service providers to gather data from the wearables and monitor the bracelets themselves. This attribute was perceived as highly advantageous in terms of facilitating efficient surveillance and guaranteeing the integrity of the sensor bracelets, hence preventing any unauthorized tampering or substitution:

I am not a technology person, but I imagine that if we are using such bracelet, I would prefer that it send me data, and I could pull data from it as well. So, push and pull material for data. In addition, the activation and deactivation are central. The user shouldn’t be able to activate or deactivate something. I need to know that someone has cut it, removed it, given to someone else or exchanged, so I can track people.

Participant 2; health care provider and public health specialist

###### Cost Reduction Through Private Sponsorship

Considering the cost of the bracelet is of utmost importance as participants emphasized the need to minimize the financial load on hajj authorities. The consensus among participants was that developing or deploying such a device within the context of hajj must strike a delicate equilibrium between cost-effectiveness and the essential functionalities it provides:

Honestly, the operation model for this whole business should be provided by the private sector, not the government.

Participant 1; systems engineering, business, and project management

###### Interest of Entities Involved

A significant aspect highlighted by participants was the identification of the interests of the entities involved in the project and assessing the value derived from integrating the technology into their road map development. Among the many organizations participating in the hajj season, such as the Ministry of Health, Ministry of Hajj and Umrah, Ministry of Interior, and others, a number of duties and responsibilities were highlighted:

The Ministry of Hajj and Umrah focuses on the quality of services, which is why we focus on measuring satisfaction with crowd management, movements, and safety. We do not focus on diseases and infectious diseases because it is different from the scope of work we are looking for. We are not wasting our effort and time on technologies of that interest. It is not our specialist as a ministry, but the Ministry of Health are working on this file.

Participant 1; systems engineering, business, and project management

###### User Motivation: Promoting the Device for Pilgrim Safety

The significance of encouraging pilgrims to adopt wearable sensor bracelets actively was emphasized by the participants. They stressed the importance of communicating a message that revolves around the person’s interest in and reasons for using the technology, especially in light of their safety. It was widely agreed upon that presenting the use of wearables during hajj as a matter of personal preference closely linked to the individual’s welfare enhanced the probability of acceptance among pilgrims. The participants expressed reservations about adopting methods that could be interpreted as invasive or limiting, emphasizing the importance of presenting wearables as instruments for individual safety and autonomy:

I think you can work it out with the Ministry of Hajj and Umrah, and the Hajj missions by agreeing that the wearable’s objective is user health and user safety. They should propose it from the health and safety aspect, so people accept wearing it.

Participant 2; health care provider and public health specialist]

#### Recommendations for Wearable Sensor Bracelets for Disease Detection During Hajj

Interviewees underlined and provided valuable insights into many prerequisites that must be considered to successfully and efficiently implement wearable sensor bracelets for disease detection during hajj.

##### Comprehensive Infrastructure

The importance of establishing a resilient and all-encompassing infrastructure to effectively handle the large number of devices deployed in the vicinity of the holy sites of Makkah and enable seamless data transmission was emphasized as a crucial component. Moreover, the emphasis was placed on the many components and characteristics of the devices that could efficiently carry out the functions of disease detection and tracking throughout the hard period of hajj considering the large number of participants:

An important variable in the success of smart cities is first the infrastructure.

Participant 4; communication engineering and AI

##### Device Characteristics and Features

#### Strength, Type, and Independency of Connectivity

The current state of connection in holy places was underscored as being in its nascent phase notwithstanding the progress made in 4G and 5G technologies and the deployment of IoT infrastructure:

In previous years, the infrastructure of internet connectivity for 4G and 5G improved remarkably. Connectivity needs to be strong around all holy sites and areas. Not all areas were 100% covered, some were covered under a certain company, and others were served under another coverage company. In addition, the large number of people in a certain area using that network could weaken the connectivity.

Participant 4; communication engineering and AI

A cost-effective communication infrastructure was suggested as a means to facilitate data transmission for devices and guarantee equitable service for the substantial number of pilgrims. The proposal entails offering complimentary access to this form of connectivity while considering the economic strain on both the organizers and the users:

There is the cost of the device itself and the cost of communication. What I mean by communication is your network package or availability of Wi-Fi services.

Participant 4; communication engineering and AI

Emphasizing the necessity for data transmission autonomy, it was critical that wearable sensor bracelets possess the capability to transmit data autonomously without dependence on an intermediary device such as a mobile phone. Autonomy is attained by the use of different forms of connectivity, such as ultrawide band:

We usually recommend that when using a smart watch not to connect it to a mobile phone, rather to a sim card or to any type of connectivity such as the UWB where data can be sent whenever it gets signal form a sensor, a device, or any connectivity.

Participant 4; communication engineering and AI

#### Accuracy and Length of Communication Range

Furthermore, the strong reliability of Bluetooth was highlighted, specifically in the context of short-range communication. Nevertheless, it was recognized that Bluetooth possesses certain restrictions, necessitating specific intervals for optimal functionality when operating over particular distances:

The advantage of Bluetooth is the strong accuracy. However, one of the limitations is that every 50-100 m you need an interval. The UWB you can reach up to 1000 meters, so less sensors.

Participant 4; communication engineering and AI

Apart from the various characteristics of the wearable sensor bracelet that were previously discussed, with a focus on accuracy and efficiency in wearable devices for disease detection during hajj, technologists emphasized the importance of battery life as a critical factor to be taken into account. The importance of selecting an appropriate connectivity option that aligns with the specific circumstances was underscored as it helps reduce excessive battery use and enables consistent and efficient use of these devices within the challenging pilgrimage setting:

Battery life as I mentioned is a factor you need to consider. How many days would you like the bracelet to last for. Nowadays companies manufacture devices based on your need, do you want it to last a day more, a week or what.

Participant 4; communication engineering and AI

##### Business Model: Selection of an Appropriate Use Case (a Methodology Used in System Analysis to Identify, Clarify, and Organize System Requirements)

Identifying the right and appropriate use case for the technology was highlighted based on user interest and needs and cost to facilitate the successful integration of new technology into the hajj system:

We need to know the appropriate and right use cases for technology usage and implementation. It needs to be clear.

Participant 4; communication engineering and AI

Participants emphasized the significance of rendering the use of wearables a personal decision, striking a balance between the device’s advantages and the costs involved. The rental model, individual activation, and targeted distribution were identified as crucial elements in attaining a more customized and economically efficient strategy for deploying wearables during the hajj pilgrimage:

A person could personally benefit from this wearable if he/she feels they have a critical condition and needs this service; it can be requested as a special request. Anyone can activate it by connecting the watch with contact numbers in an emergency. The government does not need to provide this service, especially since the watch cost is very high. We are talking about 1000 Saudi Riyal [US $266.60] for one watch. Honestly, the value for the cost was not achieved in the project.

Participant 1; systems engineering, business, and project management

##### Effective Staff Training for Wearable Implementation and Distribution

Staff training was identified as a crucial component for successfully implementing wearables. The tasks encompassed managing a substantial volume of data and guaranteeing precise allocation of the wearable devices. The hajj event yields a substantial amount of data, necessitating the provision of sufficient training to staff to handle, analyze, and understand this information effectively:

Another thing we need to think about. When I receive a large amount of data, and I am not ready for it, how can I deal with it? Last Hajj, we suddenly received a large amount of data from other departments it was very detailed/360, and we had no idea how to deal with them. We didn’t expect it.

Participant 3; health care provider and public health specialist

Another issue we need to consider is how we will guarantee the distribution of the device. You know the food itself; we have an issue with it with the campaigns. Think about this scenario. I am receiving pilgrims from various countries, and I have the bracelet to give to the campaign manager or staff that were not trained enough to match the bracelet with the right person. I can’t guarantee this; most likely, there will be a mix-up.

Participant 3; health care provider and public health specialist

##### The Need for Organic System Harmony

Participants highlighted the significance of achieving a smooth and natural incorporation of wearable devices into the system to facilitate prospective expansion. This sentiment agrees with the perspective expressed by a participant who emphasized the importance of adopting an organic approach and considering integration as a complete system. The importance of careful deliberation was emphasized by the participant, stressing the need for alignment between decision makers and persons who wear the bracelets. The primary focus lies in establishing a cohesive, interrelated framework that promotes user-friendly functionality, comprehension, and expansion:

These things need to be organic; it is a system. We need to think about the person taking the decision, the person wearing the bracelet. Everything need to be harmonised and work together. We all need to know how to deal with it and there need to be built on organic growth.

Participant 3; health care provider and public health specialist

#### Opportunities and Advantages of Wearable Sensor Bracelets During Hajj

##### Multidisciplinary Features and Distinctiveness Among Other Digital Tools With AI Integration

Participants provided insights into the possible benefits of incorporating wearables, highlighting the numerous opportunities that arise from the varied range of features offered by these devices. They emphasized the potential for transformation that is enabled by improvements in AI capabilities, which play a vital role in developing and improving these devices. The perspectives shared by the participants shed light on a favorable view on the distinctive attributes of wearable devices and the potential synergies they offer with modern AI technologies. These observations indicate a wide range of opportunities for further advancements in this field:

Yes, a Wearable sensor bracelet is a good device. Can we use it? The answer is yes. AI will take a very huge platform in public health.

Participant 2; health care provider and public health specialist

Participants underscored the unique features of wearable sensing devices compared to other gadgets such as mobile phones. The participants emphasized that wearable devices can assess a wider array of physiological indicators in contrast to mobile devices. This distinctive capacity of wearable sensors makes them valuable instruments for gathering a complete array of health-related data, surpassing typical mobile devices’ capabilities:

So, both devices; smart watches and mobile phone can use Bluetooth to track and trace infected individuals and people who have been in contact with. However, Smart watches have an advance of measuring vital signs.

Participant 4; communication engineering and AI

##### Gradual Integration of Wearables During Hajj

Participants emphasized the importance of gradually integrating wearable devices as public health tools for effective implementation into the hajj system:

...of course. Wearables started to get large space in healthcare. Yes, we can use wearables, but I wouldn’t use them for public health at first. It could start with healthcare tackers, so I use the wearable, which can give signals to the healthcare tacker first. Each group of pilgrims have their own identifier (healthcare tacker) or medical mission, so it gives a warning to the medical mission, which gives him a summary of number of cases for example 4 cases have X without a diagnosis just based on the wearable. Then, as a public health department, I get notified and decide what actions need to be taken, such as checking A, B, & C to ensure it is X or Y.

Participant 2; health care provider and public health specialist

### A Brief Summary of the Findings From the Second Group

The second group of participants cited their duty to serve the guests of God during their hajj journey to Makkah. They discussed the many types of *tawafa* organizations they belonged to (a service provider for external pilgrims) and the role of each one. *Tawafa* organizations are classified into 4 distinct groups. The first group consists of 6 organizations responsible for assisting pilgrims in Makkah and the holy sites that are segregated into subgroups according to the countries and continents from which they originate. These six organizations are for pilgrims coming from (1) Iran; (2) South Asia; (3) Turkey, Europe, Australia, and America; (4) Arabic countries; (5) African non-Arabic countries; and (6) Southeast Asia. The second group consists of 1 organization responsible for providing water to pilgrims at the Sacred House. The third group consists of an organization that is licensed to receive and dispatch pilgrims arriving via air, sea, and land ports, facilitating and arranging their departure protocols. The final group is an organization that is licensed to provide services to pilgrims visiting the sacred mosque located in Madinah city.

Several prevalent health concerns among the pilgrims they assist were brought up by the participants. The most common health problems associated with the hajj event include heat-related illnesses such as heat stroke, heart diseases, and infectious diseases. The importance of medical missions associated with each group of pilgrims was also underscored.

Participants commented on a common issue related to pilgrims’ lack of adherence to specific preventive measures designed by hajj authorities to safeguard their well-being, such as hygiene and stampedes in Jamaraat Bridge. In addition, they highlighted that the most holy site among Mina, Muzdalifah, Mount Arafat, and the Holy Mosque where the crowd density becomes high is Mina.

Participants discussed the technological improvements introduced by hajj authorities, such as mobile apps and smart cards, which provide assistance and information to pilgrims from diverse backgrounds. Nevertheless, the participants also emphasized the difficulties of integrating these technologies on a broad scale during hajj. They stressed the importance of conducting more educational campaigns to assist pilgrims in comprehending and proficiently using these technologies as well as understanding the reasons behind their deployment.

## Discussion

### Principal Findings

#### Overview

In this study, we explored participant perceptions and experiences of using wearable sensor bracelets during the hajj pilgrimage. The primary objective was to investigate the potential advantages and obstacles associated with wearables monitoring pilgrims’ well-being and detecting diseases. The main findings and themes that emerged from the interviews will be analyzed and summarized in this section to provide insights into the opportunities and challenges involved in incorporating wearable technology (sensor bracelets) into mass gatherings, including the hajj pilgrimage.

Wearable sensor bracelets have the capability to monitor the health of pilgrims and detect emerging infectious diseases during the hajj pilgrimage. However, achieving these capacities depends on properly tackling existing problems and meticulously evaluating multiple elements.

The diverse range of valuable information obtained from the interviews with participants reveals a variety of obstacles that hinder the seamless use of wearable sensor bracelets for detecting infectious diseases during the hajj pilgrimage. However, in the midst of these difficulties, a variety of factors for achieving successful implementation become apparent, uncovering hidden possibilities that these devices offer. Charting a path for future deployments in the dynamic landscape of mass gatherings such as hajj, we will unravel the multifaceted nature of these challenges, explore the promising vistas these wearables offer, and present recommendations highlighted by our interviewees’ insightful perspectives as we navigate the discussion.

#### Challenges

Numerous challenges associated with implementing wearable sensor bracelets during hajj were highlighted. The primary obstacle underlined was the anticipated opposition to technology, which may potentially emerge from providers and users of wearable devices. Participants observed that trust in technology, data privacy, information security, and human rights concerns could influence this reluctance.

The opposition of providers is affected by both the operational and financial challenges associated with implementing wearable technology into the hajj system. Although data are an essential element in detecting diseases, staff training in data collection and analysis is required to handle the anticipated volume of data produced by the devices. Participants voiced reservations about the limited and inaccurate pre-hajj data on pilgrims’ health. Furthermore, there was concern that, despite the dedicated efforts put into the implementation, the results may not correspond with the anticipated or intended value.

The issue of financial burden is not limited to providers; it affects pilgrims as well. Due to the financial obligations that pilgrims encounter during their hajj journey [36], the cost could constitute a barrier if the services were to be included in their hajj packages. The cost-related reluctance may affect users’ willingness to use the device during their hajj pilgrimage. The use of tracking and tracing methods to monitor pilgrims throughout their journey may impede their compliance and reception due to variations in their backgrounds, beliefs, ages, approaches to conducting hajj rituals, level of trust in technology, and literacy levels [3,37].

The challenges brought up by stakeholders are consistent with those identified by Yeung et al [3], especially regarding the end users in our research, who are pilgrims. The stakeholders’ worries about potential hurdles from users align with the findings of Yeung et al [3], suggesting that user resistance may stem from factors such as privacy concerns, lack of motivation, and technological fear.

The viewpoints conveyed by stakeholders and the difficulties they mentioned pertain to service providers and users and align with the concept of effort expectancy in the UTAUT model. This connection is particularly visible in the financial and operational efforts required to integrate wearables during hajj.

Data privacy is paramount while collecting user data through wearable devices in any domain. Collecting real-time data is essential for public health surveillance to diagnose infectious diseases promptly and implement appropriate interventions. Gathering sensitive data poses difficulties, especially when considering human rights and data privacy agreements. The use of wearable public health devices, particularly in mass gatherings, may be limited due to considerable challenges arising from privacy considerations.

The issue of data privacy, which is of utmost importance for wearable devices in the context of mass gatherings, is intricately linked to the difficulty of amassing data with user consent. Participants highlighted that addressing privacy concerns and securing user consent presented substantial obstacles, impeding the success of the previous smart bracelet initiative. Azodo et al [6], who discussed information privacy, data sharing, and autonomy, brought attention to this layer of complication brought about by using wearables in health care.

The stakeholders’ willingness to adopt wearable sensor bracelets for disease detection and tracking during hajj was strongly linked to the idea of performance expectancy in the UTAUT model. The stakeholders’ trust in the technology was greatly impacted by the device’s performance and its capacity to meet fundamental functions. This is consistent with the concept of performance expectancy, which highlights the perceived utility of the technology. Furthermore, assessing the device’s utility, including factors such as dependability, precision, and compatibility, highlights the significance of technology characteristics in the TTF model. These functionalities play a vital role in shaping stakeholders’ perceptions and adoption of the technology within the specific context of hajj.

#### Considerations for Promoting Acceptance and Adoption

Overcoming current obstacles that hinder the adoption and use of wearable sensor bracelets during hajj is vital. Participants emphasized important aspects that play a significant role in promoting the success of the implementation.

The interviews demonstrated that stakeholders believe that user motivation is crucial in shaping their acceptance of the device. The interviews covered several aspects that contribute to promoting acceptance. Improving data availability to enhance accessibility is a primary driver for addressing stakeholders’ needs. Furthermore, considering the interests of the parties involved in managing hajj ensures the incorporation of technology into their plans for future development in line with their particular requirements and strategies.

The wearables’ functionality and capacity to collect accurate, dependable, and real-time data were given thoughtful consideration, addressing issues brought up in the literature on the difficulty of guaranteeing wearable accuracy [11,27,38]. Increasing stakeholder trust in technology becomes increasingly challenging when it comes to the known difficulties in ensuring wearable accuracy. The need to ensure essential device functions was stressed by participants as a means of promoting wearable trust and dependence. These qualities are essential for overcoming wearable accuracy difficulties, building stakeholder trust, and influencing their choice to use technology for disease detection and hajj surveillance.

The emphasis on accuracy and different device characteristics is consistent with the concept of performance expectancy in the UTAUT. Stakeholders emphasized the necessity for features that qualify the device for successful disease diagnosis and saw performance as critical to the technology. In addition to being in line with performance expectations, addressing issues with accuracy and key features is crucial for shaping stakeholders’ opinions of the technology.

There was a unanimous consensus on the necessity of incorporating private sponsorship as a business model for providing the device. By leveraging public-private partnerships, Aina et al [24] propose resolving the obstacles linked to smartification and digitalization in urban service financing. As suggested by stakeholders, this strategy reduces the financial and operational burden on hajj stakeholders related to implementing and controlling wearable devices for disease diagnosis and tracking. The fact of private sponsorship coincides with the UTAUT’s *facilitating condition* construct, offering a favorable setting and support for the effective integration of wearable technology during hajj.

During the interviews, participants emphasized safety as a crucial factor that significantly affects the adherence to and acceptance, adoption, and use of wearable sensor bracelets by pilgrims. Having sufficient user motivation and willingness to acquire and use the wearable device is essential for acceptance [39]. Although Saudi Arabia has made significant efforts to guarantee the safety of pilgrims and control crowds to minimize the occurrence of stampedes and the spread of diseases, the difficulty remains [40].

It is widely agreed that presenting the use of wearables during hajj as a personal choice strongly linked to individual well-being greatly increases the chances of pilgrims accepting it. This is consistent with the participants’ emphasis on the significance of selecting the appropriate business model for implementing such devices. Participants also voiced apprehensions over techniques that can be interpreted as intrusive or constraining, emphasizing the significance of portraying wearables as instruments for personal safety and independence.

#### Recommendations

Several crucial recommendations were proposed to tackle the limitations of incorporating wearable sensor bracelets for disease detection during hajj. A robust IoT infrastructure emerged as a crucial element in successfully deploying the device. The type of network connectivity and its strength were highlighted as key considerations, emphasizing the capacity to handle the volume of users and data transmission coupled with the availability of free connectivity for all pilgrims.

Participants emphasized the importance of implementing wearables for public health surveillance in a gradual and systematic manner. The gradual integration focuses on and enables smooth integration by initially guaranteeing the compatibility of the technology with the surveillance system used during hajj. Compatibility is a vital element within the TTF model that describes the features of the technology.

Participants with technology experience provided valuable comments that highlighted the need to address data availability difficulties faced by hajj stakeholders. They emphasized the importance of establishing a strong, accurate health data system to resolve these challenges. The proposed solution entails using blockchain technology to establish customized electronic health records specifically designed for pilgrims. This approach is in keeping with the recommendations presented in the research by Yeung et al [3], emphasizing the capacity of blockchain technology to tackle issues concerning the secure and decentralized storage and transmission of digital data in health care infrastructure.

Participants put forth a distinctive suggestion concerning carefully considering and selecting a suitable business model to ensure the seamless integration and use of wearable sensors during the hajj ritual. Walsh [35] states that smartwatch applications and use cases differ in many fields, offering various services and functions. The wide range of differences among pilgrims during hajj and *umrah* offers a chance to create applications that can improve their overall experience. A novel proposal entails implementing a rental system for individuals who wish to use such devices during their hajj pilgrimage either for overall well-being or for specific health concerns.

#### Opportunities

Implementing wearable sensor bracelets for infectious disease detection during hajj comes with difficulty, yet the opportunities for this technology’s use are considerable.

The use of AI greatly enhances the potential of wearables in public health surveillance, representing a promising advancement. Wearable devices, which include communication modules and networking capabilities, possess modern hardware technologies. These devices can supply vital data that power AI procedures [41]. This technological advancement facilitates the uninterrupted transmission of up-to-date information without requiring direct engagement from health care staff, thereby establishing wearables as a potent and nonintrusive instrument for augmenting public health surveillance.

The capacity of wearable sensor bracelets to evaluate a wider range of physiological indications gives them a clear edge over other technologies such as mobile devices. Their ability to adapt and be used in various ways makes them very valuable in the health care industry.

Wearable sensor bracelets greatly enhance public health surveillance during hajj by carrying out functions such as disease identification, health monitoring, tracking, and tracing. They stand out from other devices because of their unique characteristics, which provide real-time health tracking, detection, and monitoring. This helps with the efficient administration of public health during hajj. This is consistent with the broader idea of the TTF model, in which wearables are customized to carry out certain duties and operations that are essential for effective public health management in the special circumstances of hajj.

The degree to which wearable sensor bracelets can fulfill the unique requirements of hajj stakeholders is one of the key elements determining the desire to use them for disease detection during hajj. The link between hajj stakeholders’ desire to use wearables for disease detection and their perception of the devices’ capabilities emphasizes the importance of performance expectancy as a critical element of the UTAUT, which affects providers’ desire to embrace the technology. Furthermore, achieving successful implementation relies on facilitating conditions, underscoring the need for a supportive environment for efficient adoption.

Furthermore, the *technology characteristics* component, as defined by the TTF model, has a crucial influence on the intention to use. The characteristics and qualities of the technology have a substantial influence on providers’ inclination to embrace it. The relationship between the UTAUT and TTF model highlights how users’ intention to adopt and use wearable sensor bracelets for disease detection during hajj is influenced by their perception of the technology’s performance, facilitating conditions, and characteristics.

### Strengths, Limitations, and Future Work

#### Strengths

This study is the first to address the opportunities and limitations of wearable sensor bracelets for hajj. It provides a thorough understanding of these issues from the perspective of key stakeholders. The thoroughness of the investigation provides useful insights into the complex dynamics surrounding the implementation of these devices.

Moreover, the combined use of the UTAUT and TTF models proved advantageous in comprehending stakeholder viewpoints comprehensively. This method enabled a thorough investigation of the possible uses of wearable devices for detecting diseases, specifically during the hajj pilgrimage.

#### Limitations

One potential limitation is that the range of stakeholders from different disciplines that were included in the interviews was not very diverse. Broadening the pool of respondents would enhance our understanding of the system and the interconnectedness between its components.

In addition, a possible drawback arises from the involvement of a single researcher in the coding and analytic process. This limited worldview may lead to the potential for misjudging participants’ perspectives. Adding numerous analysts or external assessments could have enhanced the coding process by incorporating varied perspectives and increasing its rigor. Considering the expansion of the analytical team in future studies may help limit any biases in interpreting participant replies.

#### Future Work

To optimize the efficient deployment of wearable sensor bracelets, further studies should prioritize obtaining perspectives from end users. Gaining insights into their perspectives and embracing their adoption of the device is essential for formulating tactics that align with user requirements and preferences. This would additionally enhance the successful integration and use of wearable sensor technology in pertinent contexts.

### Conclusions

The significant contribution of this work lies in its thorough investigation of crucial factors in implementing wearable sensor bracelets for the detection of infectious diseases during the hajj event. Our study used the integration of the TTF and UTAUT frameworks to better understand the adoption of wearable sensors by stakeholders. The purpose of this integration was to investigate the perceived likelihood of technology adoption during hajj and the stakeholders’ viewpoints on the compatibility between the task of disease detection and the technology used. Our aim was to integrate these theories to offer a thorough comprehension of the aspects that impact the acceptance and efficacy of wearable sensors in this particular setting. This study underscores the need to resolve crucial issues to guarantee the efficient implementation and use of wearable sensors in this specific case. Our results highlight multiple problems pertaining to suppliers, users, and technology, which impose a cost on hajj stakeholders. Significantly, the specific attributes and functionalities of the technology play a vital role in building confidence among providers when it comes to using wearables during hajj. This study highlights the importance of having the ability to access pre-hajj health data for health care providers to gain a more comprehensive understanding of pilgrims’ requirements. Although there have been some breakthroughs, it is crucial to further develop the current infrastructure to support wearables fully. Wearables stand out from other devices due to their exceptional capacity to gather vital signs, and their potential is further amplified by encouraging outcomes from AI algorithms. This study suggests choosing a suitable business model that is customized to meet the specific demands of the users to ensure a successful deployment. A crucial element of this implementation entails creating a complete infrastructure marked by a strong and reliable network capable of prompt data transfer and accommodating a large user population. Furthermore, the unique characteristics of the devices should be in line with cost-effective advantages to guarantee a sustainable implementation.

However, deploying wearable bracelets for detecting infectious diseases during hajj may face difficulties. Therefore, it is important to conduct pilot tests and thorough evaluations to ensure their usefulness before implementing them at a large scale. This prudent approach recognizes the intricacy of the hajj environment and highlights the importance of conducting evidence-based evaluations to determine the feasibility and effectiveness of the wearable technology. Notwithstanding these difficulties, there is a pervasive conviction regarding the innate capacity of these wearable devices, cultivating optimism regarding their ultimate and widespread use in the context of hajj.

## Appendix

Multimedia Appendix 1 Example topic guide for the interviews.

## Abbreviations

AI: artificial intelligence
COREQ: Consolidated Criteria for Reporting Qualitative Research
HRV: heart rate variability
IoT: Internet of Things
TTF: task-technology fit
UTAUT: unified theory of acceptance and use of technology

## Data Availability

All data generated or analyzed during this study are included in this published article and its supplementary information files.
