# Supplementary material for: Opportunities and Challenges Surrounding the Use of Wearable Sensor Bracelets for Infectious Disease Detection During Hajj: Qualitative Interview Study
Source: JMIR Form Res. 2025 Apr 8;9:e60484. doi: 10.2196/60484 (PMC12015332; doi:10.2196/60484)
Supplement: Multimedia Appendix 1 [file formative_v9i1e60484_app1.docx]

**Example Topic Guide 1**

Interview Questions for Key Stakeholders Involved in the 2021 Smart Bracelet Project

1. Interviewee’s background: Current position and years of experience.
2. Existing Surveillance System Capabilities
   1. Current Technologies and Tools
3. 2021 Smart Hajj Initiative
   1. Experience and outcomes gained from the experiment.
   2. Services included in the smart bracelet
   3. Features of the smart bracelet.
   4. Infrastructure needed for the initiative
   5. Drawbacks of the initiative.
   6. Evaluation performed for the smart bracelet.
4. Presymptomatic Detection Using Wearable Sensor Bracelet in Hajj:
   1. Current infrastructure for wearables in Hajj.
   2. Motivation of parties involved to adopt wearable devices for infectious disease detection.
   3. Pilgrims’ acceptance.
   4. Operational cost
   5. Technological and epidemiological features of the sensor bracelets.

**Example Topic Guide 2**

Interview Questions for Stakeholders at Hajj Service Providers

1. Interviewee’s background: Current position and years of experience.
2. Role of Tawafa Organization
   1. Roles and responsibilities
   2. Services offered
   3. Duration and scope of services provided
3. Critical issues and challenges encountered in previous years
   1. Health issues concerns
   2. Awareness and compliance
   3. Crowd density at Holy Sites
4. Advantages of existing Hajj technologies
